# A rare association of Guillain–Barré syndrome/Miller–Fisher syndrome overlap syndrome and Herpes Simplex Virus Type 1 infection: trigger or exacerbating factor?

**DOI:** 10.1177/17562864241297086

**Published:** 2024-12-03

**Authors:** Claudia Alberti, Nicola Molitierno, Virginia Iacobelli, Daniele Velardo, Giacomo Pietro Comi, Stefania Corti, Mosè Parisi, Elena Abati

**Affiliations:** Department of Pathophysiology and Transplantation, University of Milan, Milan, Italy; Department of Pathophysiology and Transplantation, University of Milan, Milan, Italy; Department of Pathophysiology and Transplantation, University of Milan, Milan, Italy; Neurology Unit, Department of Neuroscience and Mental Health, Fondazione IRCCS Ca’ Granda Ospedale Maggiore Policlinico, Dino Ferrari Centre, Milan, Italy; Department of Pathophysiology and Transplantation, University of Milan, Milan, Italy; Neurology Unit, Department of Neuroscience and Mental Health, Fondazione IRCCS Ca’ Granda Ospedale Maggiore Policlinico, Dino Ferrari Centre, Milan, Italy; Department of Pathophysiology and Transplantation, University of Milan, Milan, Italy; Neuromuscular and Rare Diseases Unit, Department of Neuroscience and Mental Health, Fondazione IRCCS Ca’ Granda Ospedale Maggiore Policlinico, Dino Ferrari Centre, Milan, Italy; Neurology Unit, Department of Neuroscience and Mental Health, Fondazione IRCCS Ca’ Granda Ospedale Maggiore Policlinico, Dino Ferrari Centre, Milan, Italy; Neurology Unit, Department of Neuroscience and Mental Health, Fondazione IRCCS Ca’ Granda Ospedale Maggiore Policlinico, Dino Ferrari Centre, Milan, Italy; Department of Pathophysiology and Transplantation, University of Milan, Milan, Italy

**Keywords:** case report, Guillain–Barré syndrome, Herpes Simplex Virus 1, HSV-1 encephalitis, inflammatory neuropathy, Miller–Fisher syndrome

## Abstract

Guillain–Barré syndrome (GBS) and its variants represent a spectrum of acute, immune-mediated polyneuropathies with heterogeneous clinical presentations and underlying etiologies. While infectious triggers are common precursors to these disorders, the association between viral infections and autoimmune neurological conditions remains an area of active investigation. Here, we report a case of GBS/Miller–Fisher syndrome overlap syndrome in an 80-year-old male presenting with dysarthria, dysphonia, ophthalmoplegia, areflexia, and postural instability following an upper respiratory tract infection. Cerebrospinal fluid analysis revealed the unexpected detection of herpes simplex virus type 1 DNA. Treatment with intravenous immunoglobulin therapy and acyclovir resulted in a progressive recovery of neurological symptoms. This case emphasizes the role of viral infections in differential diagnosis or as potential triggers for autoimmune neurological disorders highlighting the efficacy to addressed therapy in such complex cases.

## Introduction

Guillain–Barré syndrome (GBS) represents a spectrum of acute, immune-mediated polyneuropathies and one of the major emergencies in the neuromuscular domain. GBS is a multifaceted disorder, characterized by an array of phenotypes, electrophysiological features, and prognostic outcomes.^1^

The recognition of GBS as a spectrum of disorders with discrete, complete, and incomplete forms that sometimes overlap is critical for understanding its various clinical and electrophysiological variants and their underlying mechanisms. This spectrum includes typical presentations as well as atypical forms such as Miller–Fisher syndrome (MFS), which is characterized by ophthalmoplegia, ataxia, and areflexia, often with the presence of anti-GQ1b antibodies.^2,3^

Overlapping clinical features between GBS and MFS, particularly in cases involving limb weakness and motor nerve abnormalities, have led to the classification of GBS/MFS overlap syndromes.^1,4,5^ While a variety of noninfectious triggers such as surgical procedures, trauma, certain medications (notably immune checkpoint inhibitors), vaccinations, and systemic illnesses have been identified as antecedents or risk factors for GBS, infectious events are the predominant precursors to its clinical onset.^6,7^ This association is highlighted by the frequent occurrence of GBS following respiratory or gastrointestinal infections, with pathogens like *Campylobacter jejuni*, Cytomegalovirus (CMV), and Epstein–Barr virus (EBV) being commonly implicated.^8^ In particular, molecular mimicry between antigens on nerve endings and those carried by pathogens plays a crucial role in the pathogenesis of GBS.^9^

Despite the frequent history of an infectious antecedent, presence of a pathogen in the cerebrospinal fluid (CSF) should lead to a reevaluation of the diagnosis. There are only a few reports^10,11^ that assess co-infection in the CSF.

Given their rarity, the precise relationship between CNS infections and acute autoimmune polyneuropathies is still unclear. We hereby report a rare case of GBS/MFS overlap syndrome associated with the unexpected detection of herpes simplex virus type 1 (HSV-1) in the CSF, further highlighting the complex interplay between infectious triggers and autoimmune neurological disorders.

## Case report

An 80-year-old male presented at the emergency department with the sudden onset of dysarthria, dysphonia, and gait instability that had appeared the day before and worsened in the last few hours. He had a self-resolved flu-like episode, characterized by mild fever, sore throat, and fatigue, occurring a week before the onset of neurological symptoms. His past medical history was unremarkable, except for prostate cancer that was successfully treated with radiotherapy 4 years earlier. During the initial neurological assessment, the patient displayed a slight limitation of horizontal conjugate gaze in both directions with associated diplopia only in distance vision, moderate dysarthria with a nasal quality, likely linked to weakness of the oropharyngeal muscles, and mild postural instability requiring a wide-based stance that worsened with eye closure. The patient also experienced tingling paresthesias in the distal fingertips of his hands.

Blood tests showed normal values except C-reactive protein 1.96 mg/dL (normal value 0–0.5 mg/dL). A brain computed tomography was performed, showing no acute pathological findings. Over the following 8 h, the symptoms deteriorated, with marked dysarthria almost progressing to a state of anarthria when assuming a seated position with the head flexed forward, swiftly regressing upon returning to the supine position, likely associated with weakness of the oropharyngeal muscles. Additionally, he exhibited external ophthalmoparesis with significant limitation of ocular motility in all gaze directions and associated diplopia, initial clumsiness in fine hand movements, tingling paresthesias in the palmar regions of the hands and fingers, and lost deep tendon reflexes in ankles and in the upper limbs. No weakness in the lower limbs, defined numbness, swallowing difficulties, exertional dyspnea, or sphincter dysfunction were detected.

In the following days, the patient showed further deterioration, developing a complete ophthalmoplegia, mild bilateral facial weakness, and diffuse areflexia in all four limbs.

A diagnostic lumbar puncture was performed. CSF analysis revealed normal white blood cell count, absent red blood cells, normal protein level, and normal glucose level; a high copy number of HSV-1 DNA was detected (Table 1). Serology test results for HIV, CMV, and HSV-1 immunoglobulin G (IgG) and IgM were negative. Nerve conduction studies (NCS) performed on the fourth day after symptom onset revealed reduced recruitment in the facial muscle district and slight decreases in the compound motor action potentials of the tibial nerves, while F-waves were present in all the examined extremities (Table 2). Brain magnetic resonance imaging (MRI) with contrast revealed some non-specific T2/FLAIR hyperintensities in the periventricular and subcortical white matter, without pathological enhancement following contrast administration. In the suspicion of GBS–MFS overlap syndrome, the patient was initiated on intravenous immunoglobulin (IVIg) therapy (2 g/kg divided over 5 days) and acyclovir for HSV-1 infection (10 mg/kg every 8 h for 14 days). Clinical improvement was noted within the second week with gradual resolution of ophthalmoplegia, dysarthria, and facial paresis, along with the reappearance of deep tendon reflexes in all four limbs. Furthermore, the patient no longer reported postural instability, tingling paresthesias in the hands, and regained manual skills in the fine movements. NCS repeated on the 12th day after symptom onset revealed a slowed motor conduction velocity and increased F-wave latency recorded from the right tibial nerve, disappearance of the sensory nerve action potentials from the left ulnar and left sural nerves, while the needle electromyography (EMG) examination showed an improvement in motor unit recruitment in the left orbicularis oris muscle. These findings were consistent with a predominantly demyelinating sensory-motor polyneuropathy (Table 2).

**Table 1.** Cerebrospinal fluid analysis.

	Reference range	First lumbar puncture results	Second lumbar puncture results
Opening pressure	10–20 cmH_2_O	N/A	N/A
Color	–	Colorless	Colorless
Appearance	–	Clear	Clear
Red blood cells	Absent	0	0
White blood cells	<5/µL	2/µL	3/µL
Glucose	40–70 mg/dL	60 mg/dL	56 mg/dL
Protein	10–45 mg/dL	36 mg/dL	60 mg/dL
Herpes simplex virus 1	<250 copy/mL	2565 copy/mL	0 copy/mL

The first lumbar puncture was performed 1 day after symptom onset, while the second lumbar puncture was conducted 2 weeks after symptom onset and subsequent to IVIg therapy (2 g/kg divided over 5 days) and acyclovir treatment for HSV-1 infection (10 mg/kg every 8 h for 14 days). All laboratory investigations were conducted at the same analytical laboratory.

HSV-1, herpes simplex virus type 1; IVIg, intravenous immunoglobulin.

**Table 2.** Lower and upper limbs motor and sensory NCS.

Motor conduction studies	I	II
Right posterior tibial nerve		
Distal latency (ankle)	4.0 ms	5.3 ms
Amplitude (ankle)	4.7 mV	4.4 mV
Amplitude (knee)	3.6 mV	3.3 mV
Conduction velocity	45.7 m/s	33.9 m/s
F-wave latency	55.3 ms	60.3 ms
Left posterior tibial nerve		
Distal latency (ankle)	3.8 m/s	N/A
Amplitude (ankle)	4.0 mV	N/A
Amplitude (knee)	3.0 mV	N/A
Conduction velocity	40 m/s	N/A
F-wave latency	53.5 ms	N/A
Sensory conduction studies	I	II
Left sural nerve		
Amplitude	8 mV	Absent
Conduction velocity	52.4 m/s	
Left ulnar nerve		
Amplitude	11 mV	Absent
Conduction velocity	30.8 m/s	

First (I) and second (II) electrophysiological exams were performed 4 and 12 days after the onset of symptoms.

NCS, nerve conductions studies.

A second lumbar puncture was performed 2 weeks after symptoms onset and CSF analysis revealed a normal white blood cell count, absent red blood cells, and normal glucose levels, with a mild elevation in protein levels, HSV-1 DNA was not detected (Table 1). The detection of anti-GQ1b IgG antibodies in the serum resulted negative.

## Discussion

GBS and MFS are not distinct entities but are rather part of a spectrum of disorders whose common underlying mechanism seems to be the “molecular mimicry” between antigens located on nerve terminations and antigens carried by pathogens.^9,12^

The self-limiting flu-like symptoms experienced by our patient prior to admission may suggest an infection involving an unspecified respiratory virus, which could have initiated a primary immune response, creating a state of immune activation. Concurrently, the reactivation of HSV-1 may have further complicated the immune landscape, triggering or exacerbating the clinical picture. HSV-1 is known for its ability to establish latency in the sensory ganglia of the cranial nerves.^13,14^ Reactivation of the virus can induce both localized and systemic infections, which may precipitate a spectrum of neurological symptoms, including those affecting the cranial nerves. In the context of GBS/MFS overlap syndrome, HSV-1’s reactivation may contribute to cranial nerve dysfunction. The virus’s affinity for cranial nerves, combined with the immune system’s response to HSV-1, may exacerbate or trigger an autoimmune attack on these nerves, leading to the clinical manifestations observed in this case. Beyond cranial involvement, the immune response against both pathogens may also play a role in peripheral nerve pathology through mechanisms such as molecular mimicry.

CSF examination helps confirm the diagnosis of GBS and rule out alternative causes. The key finding is a significant increase in protein levels with a normal cell count, known as albuminocytological dissociation. This indicates increased blood–nerve barrier permeability, especially at the proximal nerve root.

Albuminocytological dissociation is absent in over half of GBS patients during the first week but appears in more than 90% by the second week. A mild increase in cell count (10–20 cells/mm^3^) occurs in up to 5% of cases, but counts exceeding 50 cells/mm^3^ suggest other conditions. In our patient, a slight increase in CSF protein was noted 2 weeks after symptom onset, with a normal cell count electrophysiology. Examinations, including NCS and needle EMG examination, are carried out according to established electrophysiological guidelines to confirm GBS diagnosis,^15–19^ rule out similar conditions, provide insights into the prognosis by distinguishing between axonal and demyelinating forms, and assess the degree and location of axonal damage. If electrodiagnostic testing performed at the initial presentation is nondiagnostic, repeat testing may be performed 1–2 weeks apart.^20,21^ The subsequent test offers a more accurate classification of the subtype and evaluates the extent of axonal damage, which is crucial for predicting the outcome. In patients diagnosed with GBS/MFS overlap syndrome, electrophysiological studies frequently reveal abnormalities in both motor and sensory nerves.^22,23^ This is in contrast to classic MFS distinguishing it from classic MFS, where the involvement is often limited to sensory nerves or may even be entirely normal.^2,24^ In the case described, clinical finding of mild bilateral facial weakness, along with electrophysiological findings included reduced recruitment in the facial muscle district, reduced motor conduction velocity, increased F-wave latency, and the disappearance of sensory action potentials, confirming both motor and sensory involvement, consistent with an overlap syndrome. Despite what is described in the literature, where brain MRI with gadolinium is an excellent confirmatory test for diagnosing MFS, there has been no observed anomalous contrast enhancement of the cranial nerves in our patient.^25,26^ However, a normal brain MRI does not exclude the diagnosis, notably in atypical cases such as MFS–GBS overlap syndrome.^27^ Anti-GQ1b is the most frequent antiganglioside antibody detected in MFS patients’ sera (approximately 85%–90% of patients). GQ1b gangliosides are located at the neuronal endings of oculomotors nerves (III, IV, and VI cranial nerves), dorsal roots of spinal cord, and fibers of neuromuscular spindles, explaining the classic symptomatic triad observed in patients. Despite anti-GQ1b antibodies are frequently associated with MFS and GBS/MFS overlap syndromes, their absence does not rule out the diagnosis.^6,9,15^ This may be due to fluctuations in antibody levels during the course of the disease. Furthermore, the sensitivity of the test can vary, and in some instances, the autoimmune response may target other gangliosides or epitopes not covered by standard testing. The clinical presentation and diagnostic tests such as CSF analysis and electrodiagnostic studies often take precedence over serological findings. In cases where the clinical features suggest GBS/MFS overlap syndrome, the absence of anti-GQ1b antibodies should not preclude the diagnosis, especially if other supportive evidence is present.

The specific treatment of GBS and its variants typically involves administering IVIg and plasma exchange therapy,^2,28–30^ resulting in gradual recovery for most patients. In the presented case, the combination of immunomodulatory therapy with IVIg along with specific antiviral therapy led to a progressive and almost complete recovery.

Bies et al.^11^ recently reported a case of a patient diagnosed with MFS with the detection of HSV-1 in the CSF following a *Campylobacter jejuni* acute infection, treated with dual intravenous therapy with immunoglobulins and acyclovir, resulting in a significant clinical improvement within the first 72 h. Although the majority of cases described in the literature regarding the rare association between HSV-1 and GBS spectrum-related syndromes present a mildly severe phenotype and respond well to dual therapy, Dilena et al.^10^’s study describes a case of a fulminant infantile GBS variant presenting as peripheral locked-in syndrome associated with HSV-1 infection with no response to acyclovir and IVIg.

## Conclusion

The presented case highlights the heterogeneous nature of GBS and its variants. It also underscores the importance of considering viral infections in differential diagnosis and as potential triggering or facilitating factors in the manifestations of autoimmune neurological disorders, such as MFS. In our patient, the overlap syndrome of GBS–MFS associated with HSV-1 detection in the CSF suggests a possible link between viral infection and the development of autoimmune pathology.

The involvement of both sensory and motor nerves in electrophysiological studies of GBS/MFS overlap syndrome provides a crucial diagnostic clue that distinguishes it from classic MFS. This distinction is essential for guiding appropriate treatment strategies, as GBS/MFS overlap syndrome may require more aggressive immunomodulatory therapy compared to isolated MFS. The recognition of these electrophysiological patterns can also help clinicians anticipate the potential for more extensive neurological impairment and adjust management plans accordingly.

Timely and targeted therapeutic management, combining IVIg immunomodulatory therapy with specific antiviral therapy led to progressive recovery of neurological symptoms in the patient. This highlights the importance of a multidisciplinary approach in treating such complex conditions. Nonetheless, there is still a dearth of literature directly correlating the potential of HSV-1 to precipitate or hasten the development of MFS.

Further research is warranted to better understand the underlying mechanisms linking viral infections to the development of autoimmune neurological disorders and to optimize treatment strategies for affected individuals.

## Declarations

**Ethics approval and consent to participate:** Ethical approval to report this case was obtained by the local Institutional Review Board (“Comitato Etico Milano Area 2 Fondazione IRCCS Ca’ Granda Ospedale Maggiore Policlinico” Milan, Italy approved the study protocol code: 239_2023). Consent to participate: Not applicable.

**Consent for publication:** Written informed consent was obtained from the patient for their anonymized information to be published in this article.

**Availability of data and materials:** Not applicable.

## References

[1] van Doorn PA Van den Bergh PYK Hadden RDM , et al. European Academy of Neurology/Peripheral Nerve Society Guideline on diagnosis and treatment of Guillain–Barré syndrome. Eur J Neurol 2023; 30(12): 3646–3674. 37814552 10.1111/ene.16073

[2] Fisher M. An unusual variant of acute idiopathic polyneuritis (syndrome of ophthalmoplegia, ataxia, and areflexia). N Engl J Med 1956; 255(2): 57–65. 13334797 10.1056/NEJM195607122550201

[3] Chiba A Kusunoki S Shimizu T , et al. Serum anti-GQ1b IgG antibody is associated with ophthalmoplegia in Miller Fisher syndrome and Guillain–Barré syndrome: clinical and immunohistochemical studies. Ann Neurol 1993; 34(6): 754–758. 10.1212/wnl.43.10.1911 8413947

[4] Govoni V Granieri E Tola MR , et al. The frequency of clinical variants of Guillain–Barré syndrome in Ferrara, Italy. J Neurol 1999; 246(11): 1010–1014. 10631631 10.1007/s004150050505

[5] Overell JR Willison HJ. Recent developments in Miller–Fisher syndrome and related disorders. Curr Opin Neurol 2005; 18(5): 562–566. 16155441 10.1097/01.wco.0000173284.25581.2f

[6] Yuki N Hartung HP. Guillain–Barré syndrome. N Engl J Med 2012; 366(24): 2294–2304. 22694000 10.1056/NEJMra1114525

[7] Hughes RA Cornblath DR. Guillain–Barré syndrome. Lancet 2005; 366(9497): 1653–1666. 16271648 10.1016/S0140-6736(05)67665-9

[8] van Doorn PA Ruts L Jacobs BC. Clinical features, pathogenesis, and treatment of Guillain–Barré syndrome. Lancet Neurol 2008; 7(10): 939–950. 18848313 10.1016/S1474-4422(08)70215-1

[9] Willison HJ Jacobs BC van Doorn PA. Guillain–Barré syndrome. Lancet 2016; 388(10045): 717–727. 26948435 10.1016/S0140-6736(16)00339-1

[10] Dilena R Strazzer S Esposito S , et al. Locked-in-like fulminant infantile Guillain–Barré syndrome associated with herpes simplex virus 1 infection. Muscle Nerve 2016; 53(1): 140–143. 26372816 10.1002/mus.24908

[11] Bies JJ Hassan M Prakash S , et al. A rare association between herpes simplex virus type 1 and Miller–Fisher syndrome. Cureus 2023; 15(4): e38163. 10.7759/cureus.38163 PMC10219615 37252484

[12] Yuki N. Infectious origins of, and molecular mimicry in, Guillain–Barré and Fisher syndromes. Lancet Infect Dis 2001; 1(1): 29–37. 11871407 10.1016/S1473-3099(01)00019-6

[13] Kennedy PGE Steiner I . Recent issues in herpes simplex encephalitis. J Neurovirol 2013; 19(5): 346–350. 23775137 10.1007/s13365-013-0178-6

[14] Steiner I. Herpes virus infection of the peripheral nervous system. Handb Clin Neurol 2013; 115: 543–558. 23931801 10.1016/B978-0-444-52902-2.00031-X

[15] Uchibori A Gyohda A Chiba A. Ca(2+)-dependent anti-GQ1b antibody in GQ1b-seronegative Fisher syndrome and related disorders. J Neuroimmunol 2016; 298: 172–177. 27609292 10.1016/j.jneuroim.2016.07.021

[16] Hadden RD Cornblath DR Hughes RA , et al. Electrophysiological classification of Guillain–Barré syndrome: clinical associations and outcome. Plasma Exchange/Sandoglobulin Guillain–Barré Syndrome Trial Group. Ann Neurol 1998; 44(5): 780–788. 9818934 10.1002/ana.410440512

[17] Shang P Zhu M Wang Y , et al. Axonal variants of Guillain–Barré syndrome: an update. J Neurol 2021; 268(7): 2402–2419. 32140865 10.1007/s00415-020-09742-2

[18] Uncini A Ippoliti L Shahrizaila N , et al. Optimizing the electrodiagnostic accuracy in Guillain–Barré syndrome subtypes: criteria sets and sparse linear discriminant analysis. Clin Neurophysiol 2017; 128(7): 1176–1183. 28521265 10.1016/j.clinph.2017.03.048

[19] Uncini A Kuwabara S. The electrodiagnosis of Guillain–Barré syndrome subtypes: where do we stand? Clin Neurophysiol 2018; 129(12): 2586–2593. 30419502 10.1016/j.clinph.2018.09.025

[20] Albers JW Donofrio PD McGonagle TK. Sequential electrodiagnostic abnormalities in acute inflammatory demyelinating polyradiculoneuropathy. Muscle Nerve 1985; 8(6): 528–539. 16758578 10.1002/mus.880080609

[21] Gordon PH Wilbourn AJ. Early electrodiagnostic findings in Guillain–Barré syndrome. Arch Neurol 2001; 58: 913–917. 11405806 10.1001/archneur.58.6.913

[22] Sekiguchi Y Mori M Misawa S , et al. How do electrophysiological studies in Fisher syndrome compare with those in Guillain–Barré syndrome? J Neurol Neurosurg Psychiatry 2012; 83(2): 194–197.

[23] Uncini A Manzoli C Notturno F , et al. Pitfalls in electrophysiological diagnosis of Guillain–Barré syndrome subtypes. J Neurol Neurosurg Psychiatry 2010; 81(10): 1157–1163. 20870864 10.1136/jnnp.2010.208538

[24] Mori M Kuwabara S Fukutake T , et al. Clinical features and prognosis of Miller Fisher syndrome. Neurology 2001; 56(8): 1104–1106. 11320188 10.1212/wnl.56.8.1104

[25] Garcia-Rivera CA Rozen TD Zhou D , et al. Miller Fisher syndrome: MRI findings. Neurology 2001; 57: 1755. 11723258 10.1212/wnl.57.10.1755

[26] Kiphuth IC Saake M Lunkenheimer J , et al. Bilateral enhancement of the cranial nerves III–XII in severe Miller–Fisher syndrome. Eur Neurol 2009; 62: 252–253. 19672080 10.1159/000232929

[27] Chaudhry V Corse A Cornblath DR , et al. MRI in the diagnosis of chronic inflammatory demyelinating polyneuropathy and related neuropathies. Muscle Nerve 2000; 23(3): 507–511.10716760

[28] Shahrizaila N Lehmann HC Kuwabara S. Guillain–Barré syndrome. Lancet 2021; 397: 1214. 33647239 10.1016/S0140-6736(21)00517-1

[29] Hughes RA Swan AV van Doorn PA. Intravenous immunoglobulin for Guillain–Barré syndrome. Cochrane Database Syst Rev 2014; 2014(9): CD002063. 10.1002/14651858.CD002063.pub6 PMC6781841 25238327

[30] Chevret S Hughes RA Annane D. Plasma exchange for Guillain–Barré syndrome. Cochrane Database Syst Rev 2017; 2(2): CD001798. 10.1002/14651858.CD001798.pub3 PMC6464100 28241090

